# Characterization of *Xenopus* Tissue Inhibitor of Metalloproteinases-2: A Role in Regulating Matrix Metalloproteinase Activity during Development

**DOI:** 10.1371/journal.pone.0036707

**Published:** 2012-05-31

**Authors:** Liezhen Fu, Guihong Sun, Maria Fiorentino, Yun-Bo Shi

**Affiliations:** 1 Section on Molecular Morphogenesis, Program in Cellular Regulation and Metabolism (PCRM), Eunice Kennedy Shriver National Institute of Child Health and Human Development (NICHD), National Institutes of Health (NIH), Bethesda, Maryland, United States of America; 2 Key Laboratory of Allergy and Immune-related Diseases and Centre for Medical Research, School of Medicine, Wuhan University, Wuhan, People‘s Republic of China; Laboratoire Arago, France

## Abstract

**Background:**

Frog metamorphosis is totally dependent on thyroid hormone (T3) and mimics the postembryonic period around birth in mammals. It is an excellent model to study the molecular basis of postembryonic development in vertebrate. We and others have shown that many, if not all, matrix metalloproteinases (MMPs), which cleave proteins of the extracellular matrix as well as other substrates, are induced by T3 and important for metamorphosis. MMP activity can be inhibited by tissue inhibitors of metalloproteinase (TIMPs). There are 4 TIMPs in vertebrates and their roles in postembryonic development are poorly studied.

**Methodology/Principal Findings:**

We analyzed the TIMP2 genes in *Xenopus laevis* and the highly related species *Xenopus tropicalis* and discovered that TIMP2 is a single copy gene in *Xenopus tropicalis* as in mammals but is duplicated in *Xenopus laevis*. Furthermore, the TIMP2 locus in *Xenopus tropicalis* genome is different from that in human, suggesting an evolutionary reorganization of the locus. More importantly, we found that the duplicated TIMP2 genes were similarly regulated in the developing limb, remodeling intestine, resorbing tail during metamorphosis. Unexpectedly, like its MMP target genes, the TIMP2 genes were upregulated by T3 during both natural and T3-induced metamorphosis.

**Conclusions/Significance:**

Our results indicate that TIMP2 is highly conserved among vertebrates and that the TIMP2 locus underwent a chromosomal reorganization during evolution. Furthermore, the unexpected upregulation of TIMP2 genes during metamorphosis suggests that proper balance of MMP activity is important for metamorphosis.

## Introduction

Matrix metalloproteinases (MMPs) are Zn^2+^ dependent extracellular or membrane-bound proteinases that can cleave protein components of the extracellular matrix (ECM) as well as non-ECM proteins with overlapping substrate specificities [1]–[7]. They can affect cell fate and behavior by remodeling the microenvironment surrounding the cells and/or altering inter-cellular signaling.

The activities of MMPs can be regulated at multiple levels. MMPs are synthesized as pre-enzymes. The propeptide, which targets an MMP for secretion, is cleaved upon insertion into the plasma membrane or secretion. For some MMPs, such as stromelysin 3 (ST3, also known as MMP11) and membrane type MMPs (MT-MMPs), the resulting secreted or membrane-bound form is active due to the intracellular removal of the propeptide domain by furin, a Golgi enzyme [5], [8]. For most of the MMPs, however, the secreted or membrane-bound form remains latent due to the presence of a propeptide and the cleavage of the propeptide offers a means of regulating MMP activity [1], [4], [6], [9], [10]. In addition, MMP activity can be inhibited by the naturally occurring inhibitors: tissue inhibitors of metalloproteinase (TIMPs).

There are four TIMP genes (TIMP1-4) in mammals and these TIMPs have overlapping specificities against different MMP targets [11]–[13]. Aside from functioning as MMP inhibitors, TIMPs can also have MMP-independent functions to affect cell signaling [14]. Additionally, TIMP2 can also function with MT1-MMP to activate pro-gelatinase A (MMP2) [6], [15]–[17].

We have been studying the role of MMPs during vertebrate development by using *Xenopus* metamorphosis as a model. Frog metamorphosis is totally dependent on thyroid hormone (T3) and resembles the postembryonic period around birth in mammals [18]. During metamorphosis, essentially all tissues/organs undergo T3-dependent changes. These include complete absorption of the gill and the tail, *de novo* generation of the hindlimb, and remodeling of most other organs such as the intestine. Numerous studies have shown that the metamorphic effects of T3 are mediated by T3 receptors (TRs) [19], [20]. TRs regulate the so-called direct T3 response genes at the transcription level and these genes in turn affect the expression of downstream T3 response genes. Many T3 response genes have been isolated and characterized over the years. Some of the earliest identified T3 response genes included *Xenopus (X.) laevis* ST3 (MMP11) and collagenase 3 (MMP13), and *Rana catesbeiana* collagenase 1 (MMP1) [21]–[24]. Subsequent studies have found that essentially all MMPs analyzed so far are upregulated by T3 in at least some organs/tissues during metamorphosis [21]–[37]. Some, like ST3 and MMP9-TH (T3-induced MMP9) in *X. laevis* and collagenase 1 in *Rana catesbeiana* are regulated directly by TR binding to their promoter regions [32], [38]–[40] while others are likely indirectly regulated by T3. Furthermore, *in vitro* and *in vivo* studies suggest that at least several MMPs, including ST3, gelatinase A (MMP2), and MT1-MMP, are important for tissue resorption and/or larval cell death during metamorphosis [22], [30], [41]–[44].

To investigate whether MMP activities can also be regulated by TIMPs during metamorphosis, we have characterized the *Xenopus* TIMP2 genes. We focus on TIMP2 as it can inhibit ST3 [45] and is also involved in the activation of gelatinase A (MMP2) by MT1-MMP [6], [15]–[17]. We show here that TIMP2 is a single copy gene in *Xenopus tropicalis* as in mammals but is duplicated in *Xenopus laevis*. Interestingly, the TIMP2 locus in *X. tropicalis* genome is distinct from that in human, suggesting a chromosome reorganization during evolution. Furthermore, we show that both copies of the TIMP2 genes are similarly but unexpectedly upregulated during metamorphosis in different organs that temporally correlates organ specific metamorphic changes. Our findings suggest that proper control of MMP activity is important for temporal regulation of tissue metamorphosis.

**Table 1:** Primers for RT-PCR and qRT-PCR.

Targeted gene	Forward primer	Reverse primer	Probe
ST3	5′- AGAGCCAGAGCCACAGACAAAG-3′	5′- GCAGGTAGAAGGATGAGGAGATGC-3′	N/A
TIMP2	5′-CCTTCTGCTCCCTGTTGCTATG- 3′	5′-CCCATTGTCCACTTCTTTTCCAG-3′	N/A
TIMP2A	5′-CTATGCTGTTGTTGATATCA- 3′	5′- CACGATGAAGTCACACAGGA- 3′	N/A
TIMP2B	5′-CTATGCTGTTGTTGATATGG-3′	5′-CTCAATGAAGTCACAAAGAG-3′	N/A
H4	5′-CGGGATAACATTCAGGGTATCACT-3′	5′-ATCCATGGCGGTAACTGTCTTCCT-3′	N/A
RpL8	5′-CGTGGTGCTCCTCTTGCCAAG-3′	5′-GACGACCAGTACGACGAGCAG-3′	N/A
TIMP2 for qPCR	5′-GGAGATGATGAGCTTTGGACACTA-3′	5′-GCACGATGAAGTCACACACAGGATTA-3′	5′-ATGCATCTTTCCATCACCATC-3′
RpL8 for qPCR	5′-AGAAGGTCATCTCATCTGCAAACAG-3′	5′-CAATACGACCACCTCCAGCAA-3′	5′CAACCCCAACAATAGCT-3′

## Experimental Procedures

### Animals

Wild-type frogs and tadpoles of *X. laevis* were purchased from Nasco (Fort Atkinson, WI, USA). The animal maintenance and usage for this study was approved by the Animal Use and Care Committee of Eunice Kennedy Shriver National Institute of Child Health and Human Development. Stage 54 *X. laevis* tadpoles were treated with 10 nM T3 for indicated number of days at 18°C.

### DNA and Protein Sequence Comparison

DNA and protein sequences were retrieved from GenBank. Alignment of multiple DNA or protein sequences was performed with MacVector (Accelrys Inc., San Diego, CA). *X. tropicalis* TIMP2 genomic locus, gene structure and its genomic context were identified on the National Center for Biotechnology Information (NCBI) gene database for *X. tropicalis* (Build 1.1, http://www.ncbi.nlm.nih.gov/projects/mapview/maps.cgi?taxid=8364&chr=Un|NW_003163355.1&MAPS = cntg,genes&query = uid(-2146758958)&QSTR = TIMP2&cmd = focus&fill = 40). The locus and gene structure of human TIMP2 (Gene ID: 7077), mouse TIMP2 (Gene ID: 21858) and chicken TIMP2 (Gene ID: 374178) were obtained from the NCBI gene database (http://www.ncbi.nlm.nih.gov/) under Homo sapiens Build 37.3, Mus musculus Build 37.2 and Gallus gallus Build 3.1, respectively.

**Figure 1:**
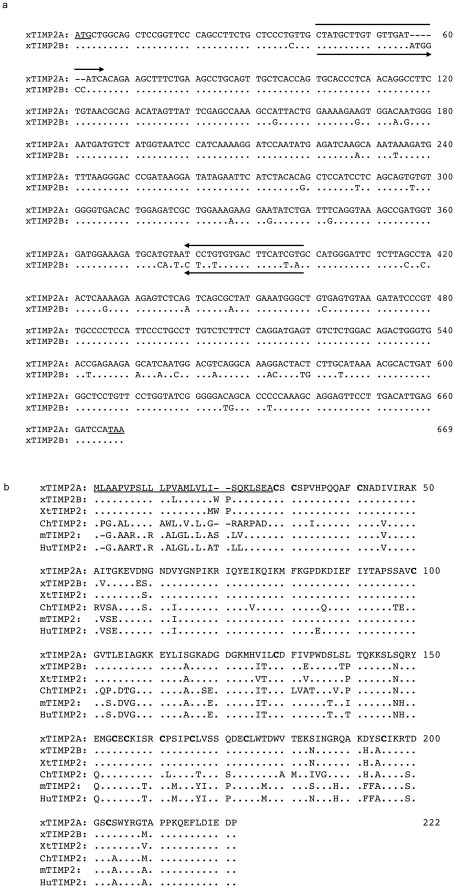
There are two *X. laevis* TIMP2 genes that are highly homologous to the TIMP2 gene in other vertebrates. A) Nucleotide sequence comparison of *X. laevis* TIMP2A and TIMP2B. Dot (.): identical sequence; dash (-):a gap introduced for better alignment. Arrowed lines indicate the locations of the primers used to distinguish the two isoforms in RT-PCR analysis. B) Comparison of the protein sequences of putative *X. laevis* TIMP2A (XlTIMP2A, AAH74452), *X. laevis* TIMP2B (XlTIMP2B, NP_001087748), and *X. tropicalis* TIMP2 (XtTIMP2, NP_001015760) with their ortholog from chick (ChTIMP2, NP_989629), mouse (mTIMP2, P25785) and human (HuTIMP2, NP_003246). The conserved cysteine sequences are in bold and the signal peptide sequence is underlined. Dot (.): identical sequence; dash (-): a gap introduced for better alignment.

**Table 2:** Homology of X. laevis TIMP2 with the ortholog of other species.

	XlTIMP2A	XlTIMP2B	XtTIMP2	HuTIMP2	mTIMP2	ChTIMP2
XlTIMP2A		**92.7%**	**91.6%**	**72.9%**	**73.2%**	**71.0%**
XlTIMP2B	92.7%		**93.3%**	**71.6%**	**72.3%**	**73.1%**
XtTIMP2	94.5%	95.9%		**73.2%**	**72.5%**	**71.6%**
HuTIMP2	75.5%	78.2%	78.2%			
mTIMP2	77.3%	78.6%	78.6%			
ChTIMP2	73.2%	77.3%	75.9%			

GenBank accession number for nucleotide sequences: HuTIMP2: NM_003255; mTIMP2: X62622; ChTIMP2: NM_204298. The homology among the nucleotide sequences (coding sequences) was on grey background.

### Reverse Transcription-polymerase Chain Reaction (RT-PCR) and Quantitative RT-PCR (qRT-PCR)

Total RNA was isolated from the tadpole intestines, hindlimbs and tails with TRIzol reagent (Invitrogen, Carlsbad, CA) and made DNA-free with RNase-free DNase I treatment. RT-PCR and qRT-PCR were performed with the primers listed below. For RT-PCR, 0.1 µg of total RNA was subjected to One-Step RT-PCR analysis with the primers specific for stromelysin 3 (ST3), both TIMP2A and B (TIMP2), TIMP2A only (TIMP2A) or TIMPB only (TIMP2B), respectively, as recommended by the manufacturer (Invitrogen, Carlsbad, CA). A pair of primers specific for histone H4 (H4) [46] or ribosomal protein L8 (RpL8) [47] were included in the RT-PCR reactions as RNA loading controls as previously described [44], [48]. The RT-PCR products were resolved on 2% agarose gels, visualized with ethidium bromide staining under UV lights and recorded with a Kodak imagining system (Gel Logic 100 Imagine System, Kodak, New Haven, CT). For qRT-PCR, first strand cDNA was prepared from 1 µg of total RNA using the Applied Biosystems High Capacity cDNA Archive kit, resulting in 20 µl cDNA solution. 2 µl of each cDNA was subjected to quantitative PCR analysis with primers/probe sets (Table 1) synthesized through Assay-by-Design (Applied Biosystem Inc.) as previously described [43]. Mixed total RNA isolated from tadpoles of all stages encompassing the whole metamorphosis was reverse-transcribed and serially diluted to generate standard curve for each qPCR assay. All the qRT-PCR analyses were carried out with a qPCR machine (Model 7000 Sequence Detection System, Applied Biosystem Inc.). A set of primer/probe set specific for rpL8 (Table 1) was used as a control for RNA input of each sample and the expression level of the gene-of-interest in each sample was normalized to that of rpL8. The RT-PCR experiments were repeated at least twice on two independent sets of RNA preparations with similar results and the qRT-PCR experiments were done on 3 sets of independent RNA preparations from pools of at least 3 tadpoles at the indicated stages or after T3 treatments. The qRT-PCR data were subjected to One-way ANOVA analysis and post-test with Turkey Method to compare all pairs.

**Figure 2:**
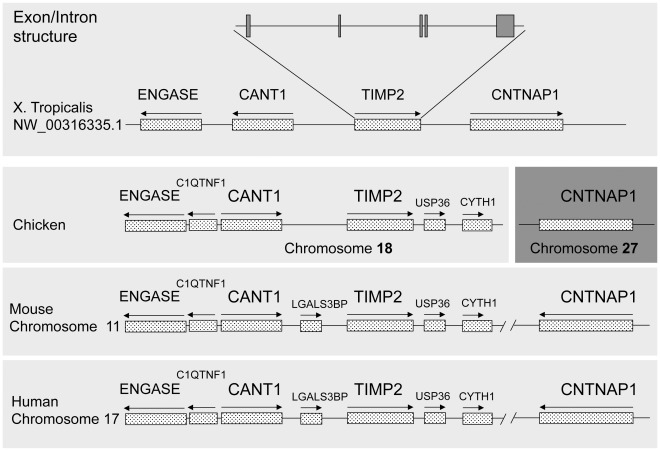
Comparison of the genomic locus and organization of *X. tropicalis* and human TIMP2. *X. tropicalis,* chicken, mouse and human TIMP2 genomic organizations were obtained from NCBI database. The lines represent the genomic sequences and the boxes with arrowed lines on top represent transcribed regions and the direction of the transcription. The lines and boxes were not drawn to the same scale for better visualization. The TIMP2 gene in all four species has the same intron/exon organization with 5 exons total as shown for *X. tropicalis* TIMP2 (not shown). Note that mouse and human TIMP2 loci are identical. In the chicken, CNTNAP1 gene is present on a different chromosome (shown in a darker shade).

**Figure 3:**
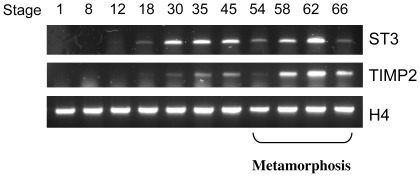
TIMP2 expression during *X. laevis* development. Total RNA was isolated from whole animals during *X. laevis* development and subjected to One-Step RT-PCR analysis with a pair of primers amplifying both TIMP2A and TIMP2B (TIMP2) or stromelysin-3 (ST3). The expression of the house-keeping gene histone H4 was similarly determined as a control.

## Results

### TIMP2 is Duplicated in X. Laevis but is a Single Copy Gene in X. Tropicalis

*X. laevis* TIMP2 was initially cloned as a 291nt fragment (GenBank accession #AY037944) encoding a 97 amino acids (aa) of peptide which was 68% homologous to the N-terminal sequences of human TIMP2 protein [30]. BLAST search with this cDNA sequence identified two additional homologous cDNA sequence entries in *X. laevis* (GenBank accession #BC074452 and NM_001094279, 99% and 94% identical to AY037944, respectively) and one in *X. tropicalis* (GenBank accession # NM_001015760, 93% identical in nucleotide sequences to AY037944) (Fig. 1a and Table 2). Both *X. laevis* clones contain a complete open reading frame (ORF) each encoding proteins of 222 aa or 220 aa, respectively, which are highly homologous to the amino acid sequence of human TIMP2, reflecting duplicated TIMP2 genes in the *X. laevis* genome. Thus, BC074452 was designated as *X. laevis* TIMP2A and NM_001094279 was designated as *X. laevis* TIMP2B. In the highly related *Xenopus* species, *X. tropicalis*, a single TIMP2 gene was found. *X. tropicalis* TIMP2 is highly homologous to both *X. laevis* TIMP2 genes at either the amino acid and nucleotide sequence levels, sharing over 90% homology (Fig. 1b and Table 2). In addition, *X. laevis* TIMP2A and TIMP2B as well as *X. tropicalis* TIMP2 share over 70% identity with human, mouse and chicken TIMP2 proteins (Fig. 1b and Table 2), though they are slightly diverged at nucleotide sequence level (Table 2). Both of the duplicated *X. laevis* TIMP2 genes and the *X. tropicalis* TIMP2 gene contain the signal peptide at the N-terminus and the conserved 8 cysteine residues, just like the TIMP2 protein in other species (Fig. 1B), suggesting a conserved function of these proteins.

Sequence search revealed that TIMP2 (Gene ID: 548477) was located in *X. tropicalis* Scaffold NW_003163355.1 (NW_003163355.1: 1397015-1429418, forward) (Fig. 2). Upstream of *X. tropicalis* TIMP2 locus on the same scaffold is *X. tropicalis* calcium activated nucleotidase 1 gene (CANT1, GenBank accession #: NM_203609), which is encoded on the reverse strand (NW_003163355.1:1379720-1387064). *X. tropicalis* contactin associated protein 1 (CNTNAP1, GenBank accession #: NM_0011097290) is located downstream of the TIMP2 gene on the forward strand (NW_003163355.1:1439806.1498859) (Fig. 2). Interestingly, while these same two genes are also present near the human TIMP2 gene on chromosome 17 (Chr.17q25), the organization is quite different between human and frog (Fig. 2). The human ubiquitin specific peptidase 36 (USP36) is located downstream of TIMP2 and the human lectin, galactoside-binding, soluble 3 binding protein (LGALS3BP) are located upstream of TIMP2, preceded by the human CANT1 gene (Chr. 17: 76886662-76899283) on the opposite strand as the CANT1 gene in *X. tropicalis* (Fig. 2). The human CNTNAP1 gene is located on the same chromosome (Chr.17: 40834632-40852010) but is far away from the TIMP2 gene and on the opposite strand as the CNTNAP1 gene in *X. tropicalis* (Fig. 2). Thus, there was a major chromosomal reorganization during evolution at either human or *Xenopus* TIMP2 locus or both. To investigate this further, we also compared the TIMP2 locus in chicken and mouse. As shown in Fig. 2, the mouse locus is identical to that in human. Interestingly, the chicken TIMP2 and CNTNAP1 gene are located on two different chromosomes, although the other side of TIMP2 locus in chicken is the same as in mammals. These results further support that a chromosomal change around the TIMP2 locus prior to the separation of human and mouse.

**Figure 4:**
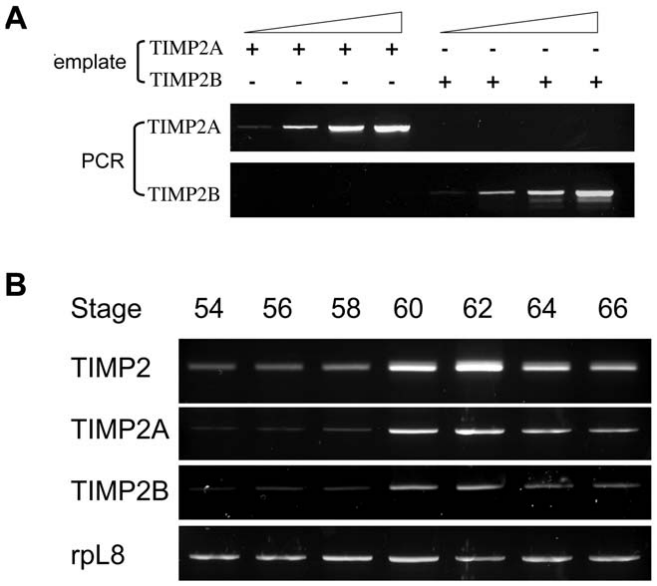
Co-regulation of TIMP2A and TIMP2B expression during intestinal metamorphosis. A) Validation of primer specificity for *X. laevis* TIMP2A or TIMP2B. The TIMP2A and TIMP2B cDNA were cloned into pCR2.1 vector, sequenced and serially diluted to serve as template DNA for PCR. Note that each primer pair specifically amplified only the intended TIMP2 gene. B) Total RNA was isolated from intestines of tadpoles during *X. laevis* metamorphosis and subjected to One-Step RT-PCR with a pair of primers recognizing both TIMP2 genes (TIMP2), specific for either TIMP2A or TIMP2B, respectively. The expression of the house-keeping gene rpL8 was similarly determined as a control.

**Figure 5:**
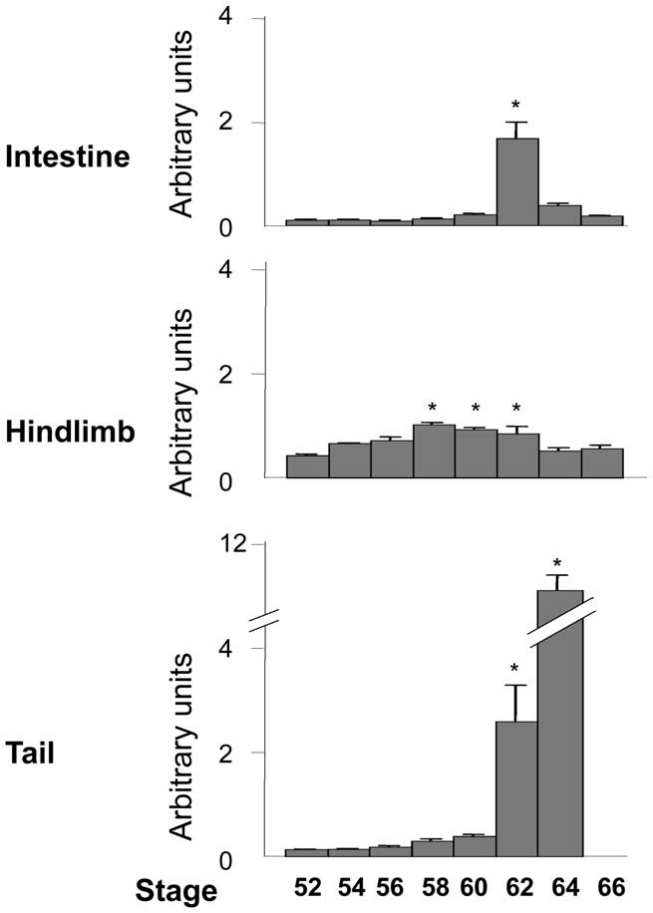
Tissue-specific regulation of TIMP2 expression correlates with metamorphosis. Total RNA was isolated from intestines, hindlimbs and tails of metamorphosing tadpoles at the indicated stages and subjected to qRT-PCR analyses with a primer/probe set specific for TIMP2 or rpL8 (RNA loading control), respectively, and the TIMP2 expression level was normalized to that of rpL8 expression level and plotted in arbitrary units. *: p<0.05 when compared to stage 52.

### Biphasic TIMP2 Expression During X. Laevis Development

To study the involvement of TIMP2 during development, we first analyzed its expression in whole animals at different stages of *X. laevis* development. For comparison, we also analyzed the expression of ST3, a well-studied MMP that can be inhibited by TIMP2 [45] . RT-PCR analysis using a primer set recognizing both TIMP2A and TIMP2B in *X. laevis* showed that like ST3, TIMP2 had little expression in eggs and was upregulated around stage 18, neural groove stage, during embryogenesis. We observed high mRNA levels around hatching at stages 35/36. After the free-living tadpole is formed by stage 45 and during the premetamorphic period stages 45–56), both TIMP2 and ST3 expression was downregulated. The second wave of expression came after stage 58 as metamorphosis begins, with peak levels of expression at the climax of metamorphosis (stage 62) (Fig. 3). Thus, TIMP2 is temporally co-expressed with one of its targets, ST3, throughout frog development and it is likely involved in organogenesis during both embryonic development and metamorphosis.

**Figure 6:**
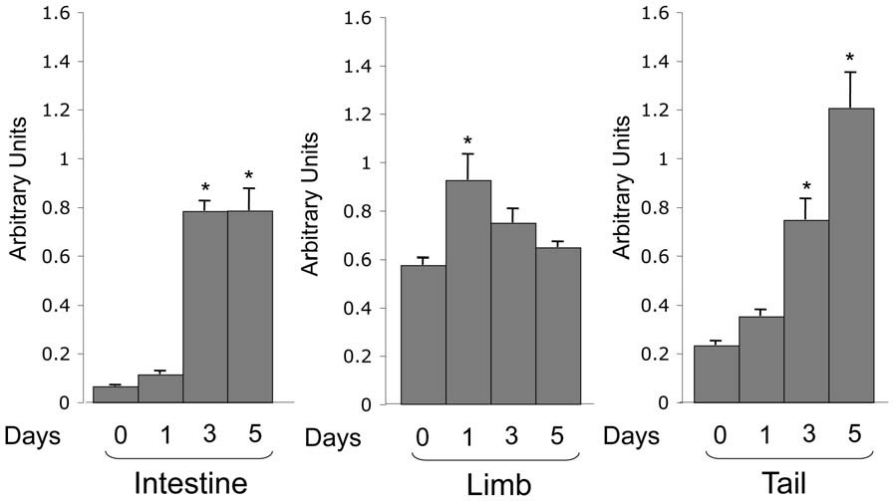
T3-inducktion of TIMP2 expression in different tissues of premetamorphic tadpoles. Tadpoles at premetamorphic stage 54 were treated with 10 nM T3 at 18°C and collected every other day for isolating total RNA from different tissues. The RNA was analyzed by qRT-PCR analysis as in Fig. 5. *: p<0.05 when compared to the group without T3 treatment.

### Temporal Regulations of TIMP2 Expression Correlates with Organ-specific Metamorphosis

The upregulation of TIMP2 expression during metamorphosis suggests a role of TIMP2 in this process. As different organs undergo changes at distinct metamorphic stages, we investigated whether TIMP2 expression is temporally correlated with metamorphosis in different organs. We chose three organs for the study. These were the limb, which undergoes morphogenesis at early stages of metamorphosis (stage 54–56) but subsequently mostly growth; the intestine, which involves the apoptotic degeneration of essentially all larval epithelial cells from stages 58–62 and de novo development of adult epithelium (stage 60–66); and the tail, which is completely resorbed during metamorphosis (mostly between stages 62–66) [18].

As there are two TIMP2 genes in *X. laevis*, we first determine whether both of the duplicated TIMP2 genes were similarly regulated, we designed gene-specific primer sets for RT-PCR analysis (Table 1). As shown in Fig. 4, the primers specifically amplified the desired target. When the expression of the TIMP2 genes was analyzed in the intestine during metamorphosis, both TIMP2A and 2B showed an identical expression pattern, highly upregulated by stage 60 and 62 (climax of metamorphosis) and subsequently reduced to a basal level toward the end of metamorphosis (stage 66). Similar results were obtained when total TIMP2 expression was analyzed with the primer set recognizing both genes, suggesting that both of the duplicated TIMP2 genes in *X. laevis* are regulated similarly to participate in organ specific changes during metamorphosis (and thus we only analyzed both TIMP2 genes together subsequently).

Next, we carried out qRT-PCR analysis with a primer/probe set recognizing both the duplicated TIMP2 genes in *X. laevis* (Table 1) on RNA from different organs at different stages of metamorphosis (Fig. 5). The results revealed that TIMP2 had little expression during early stages of intestinal metamorphosis but highly upregulated by stage 62 when larval cell death was essentially complete, in agreement with the semi-quantitative RT-PCR analysis in Fig. 4. In the limb, TIMP2 was expressed at moderate levels during premetamorphosis and was upregulated slightly after stage 56 when limb morphogenesis was largely complete (Fig. 5). Finally, in the tail, TIMP2 was highly upregulated by stage 62 (Fig. 5). These findings suggest that TIMP2 participate in organ specific changes during metamorphosis.

### TIMP2 is Induced by T3 During Metamorphosis

T3 is the causative agent of metamorphosis and can induce precocious metamorphosis when added to the rearing water of premetamorphic tadpoles. To investigate the expression pattern of TIMP2 during T3-induced metamorphosis, we treated premetamorphic tadpoles at stage 54 with T3 for up to 5 days and analyzed the expression of TIMP2 in the intestine, limb, and tail by using qRT-PCR with a primer/probe set recognizing both duplicated TIMP2 genes. As shown in Fig. 6, in the intestine, T3 was upregulated after 3–5 days of treatment. We did not see a down regulation after the induction, presumably because the 5 day treatment was not sufficient to induce complete intestinal remodeling as observed by stage 66 during natural metamorphosis. In the limb, TIMP2 was slightly induced after 1 day of treatment and dropped to lower levels subsequently (Fig. 6), mimicking the pattern during natural metamorphosis (Fig. 5). Finally, in the tail, TIMP2 was also induced strongly after 3–5 days of T3 treatment (Fig. 6), resembling the upregulation during natural metamorphosis (Fig. 5). Thus, during both natural and T3-induced metamorphosis, TIMP2 expression correlated with metamorphic changes.

## Discussion

The ECM serves as the structural scaffold for various organs/tissues and functions as a regulator of cell fate and behavior. It can function by signaling cells directly via cell surface receptors or indirectly by regulating cell–cell interactions and the availability of various factors stored in the ECM [49]–[51]. MMPs can affect these signaling processes by cleaving or remodeling the ECM. In addition, MMPs can also affect cells by cleaving non-ECM proteins, such as growth factor precursors and cell surface receptors [2], [5], [17], [52], [53]. These properties enable MMPs to influence development and pathogenesis in vertebrates and at the same time argue for a tight regulation of MMP function during development and in adult animals. In addition to the regulation of MMP expression during development and under normal or pathological conditions, MMP activity can be regulated by the expression of various TIMPs. Here, by using *Xenopus* development as a model, we showed here that TIMP2 had a biphasic expression pattern that implicated a role in both embryogenesis and metamorphosis. In addition, we observed that TIMP2 was somewhat surprisingly expressed in similar temporal patterns as one of its target, ST3, in whole animals or individual organs during metamorphosis, suggesting that a tight control of MMP function is critical for temporal regulation of organ metamorphosis.

During frog development, TIMP2 appears to be first involved in late embryogenesis, during the period of extensive organ development and maturation as the embryo prepares for free living as a tadpole. Interestingly, this embryonic expression coincides with the expression of MMPs such as ST3 [24], [41], [54]. It has been shown that mammalian TIMP2 can inhibit a number of MMPs, including MT1-MMP and ST3 [45]. The duplicated *X. laevis* TIMP2 proteins as well as the *X. tropicalis* TIMP2 protein are highly homologous with TIMP2 proteins of others species, all containing the 8 important, highly conserved cysteine residues (Fig. 1b). These suggest a similar molecular and biochemical function of TIMP2 from different species. Most MMPs except the 6 membrane type-MMPs are secreted proteins that function mainly in the extracellular space [55]. While it is difficult to localize the secreted MMPs and TIMPs, it is believed that the secreted TIMP2 should be able to inhibit MMPs in the extracellular space even if they are not expressed by the same cells. Thus, the embryonic expression pattern of TIMP2 suggests that both MMPs and TIMPs are required to ensure proper MMP activity during embryonic organ development. Once embryogenesis is completed, both ST3 and TIMP2 are downregulated. This is likely reflecting the fact that the animal now undergoes mainly growth with little organ/tissue remodeling and thus does not need MMPs or TIMPs to regulate ECM remodeling that normally accompanies such changes.

Subsequently, as the animals undergo metamorphosis, extensive organ/tissue remodeling takes place. It is not surprising that essentially all MMPs analyzed to date are upregulated at least in some organs at some developmental stages [Mathew, 2010 #3466] [56] as they likely participate in the remodeling and removing of the ECM especially where apoptosis of the larval cells takes place. Surprisingly, our studies here indicate that TIMP2 is also upregulated during metamorphosis, consistent with earlier analyses based on a partial cDNA sequence [30], [57]. These results suggest that a proper balance of MMP and TIMP activities is critical to temporally coordinate tissue-specific metamorphic transformations. Alternatively but not necessarily mutually exclusively, the upregulation of TIMP2 during metamorphosis may function to facilitate the activation of pro-MMP2 by MT1-MMP into mature MMP2, where two MT1-MMP molecules and one TIMP2 are required for the activation of one pro-MMP2 [6], [15]–[17]. This possibility is also supported by our earlier findings that *X. laevis* MT1-MMP is capable of activating pro-MMP2 in developing embryos and that MMP2 is similarly regulated during metamorphosis as TIMP2 and MT1-MMP [35], [44]. 

TIMP2 is a single copy gene in most animal species, including *X. tropicalis*. In *X. laevis*, there are two TIMP2 genes, consistent with the pseudo-tetraploid genome of this species. At both amino acid and DNA sequence levels, TIMP2 is highly conserved throughout vertebrates, suggesting that it is highly conserved evolutionally. Surprisingly, the TIMP2 genomic locus is distinct from that in human, suggesting a reorganization of the locus in either *X. tropicalis* or human during evolution, despite the preservation of the primary coding sequences. It remains to be determined if and what functional significance this may have on TIMP2 function in the two species.

In conclusion, we have shown here that TIMP2 is highly conserved and that a genomic reorganization in the TIMP2 locus occurred in either *Xenopus* or human genome during evolution. More importantly, we have demonstrated here that the two duplicated *X. laevis* TIMP2 genes are coregulated tissue-specifically during metamorphosis and their temporal expression profiles in different organs mimic those of MMPs that TIMP2 inhibits. These findings suggest that tight regulation of MMP activities is crucial for temporal coordination of the metamorphic transformations in different organs.
